# Selecting an Ecological Momentary Assessment Platform: Tutorial for Researchers

**DOI:** 10.2196/51125

**Published:** 2024-01-04

**Authors:** Lauren M Henry, Eleanor Hansen, Justin Chimoff, Kimberly Pokstis, Miryam Kiderman, Reut Naim, Joe Kossowsky, Meghan E Byrne, Silvia Lopez-Guzman, Katharina Kircanski, Daniel S Pine, Melissa A Brotman

**Affiliations:** 1 Emotion and Development Branch, National Institute of Mental Health Bethesda, MD United States; 2 Department of Anesthesiology, Critical Care and Pain Medicine Research Boston Children’s Hospital, Harvard Medical School Boston, MA United States; 3 The School of Psychological Sciences Tel-Aviv University Tel-Aviv Israel

**Keywords:** ecological momentary assessment, methodology, psychology and psychiatry, child and adolescent, in vivo and real time

## Abstract

**Background:**

Although ecological momentary assessment (EMA) has been applied in psychological research for decades, delivery methods have evolved with the proliferation of digital technology. Technological advances have engendered opportunities for enhanced accessibility, convenience, measurement precision, and integration with wearable sensors. Notwithstanding, researchers must navigate novel complexities in EMA research design and implementation.

**Objective:**

In this paper, we aimed to provide guidance on platform selection for clinical scientists launching EMA studies.

**Methods:**

Our team includes diverse specialties in child and adolescent behavioral and mental health with varying expertise on EMA platforms (eg, users and developers). We (2 research sites) evaluated EMA platforms with the goal of identifying the platform or platforms with the best fit for our research. We created a list of extant EMA platforms; conducted a web-based review; considered institutional security, privacy, and data management requirements; met with developers; and evaluated each of the candidate EMA platforms for 1 week.

**Results:**

We selected 2 different EMA platforms, rather than a single platform, for use at our 2 research sites. Our results underscore the importance of platform selection driven by individualized and prioritized laboratory needs; there is no single, ideal platform for EMA researchers. In addition, our project generated 11 considerations for researchers in selecting an EMA platform: (1) location; (2) developer involvement; (3) sample characteristics; (4) onboarding; (5) survey design features; (6) sampling scheme and scheduling; (7) viewing results; (8) dashboards; (9) security, privacy, and data management; (10) pricing and cost structure; and (11) future directions. Furthermore, our project yielded a suggested timeline for the EMA platform selection process.

**Conclusions:**

This study will guide scientists initiating studies using EMA, an in vivo, real-time research tool with tremendous promise for facilitating advances in psychological assessment and intervention.

## Introduction

### Overview

Ecological momentary assessment (EMA), a tool for collecting naturalistic data in real time, has been used in psychological science for decades [1]. In the past 10 years, the use of EMA in published scientific research has increased by 168% (Multimedia Appendix 1 [2]). Surging interest is justifiable; for example, EMA can enhance ecological validity and support the examination of within-person dynamics [1]. EMA delivery methods have evolved with technological advances [3], and leveraging mobile devices garners additional benefits, including greater accessibility and convenience, increased measurement precision, and an opportunity for the integration of passive sensing [3-5]. A proliferation of developers and platforms must be navigated to select suitable equipment [5-8]. Here, we provide guidance on platform selection for researchers launching EMA studies.

### What Is EMA?

The term EMA was coined in the 1990s to describe “...monitoring and sampling strategies to assess phenomena at the moment they occur in natural settings...” [9]. EMA is an idiographic approach. In contrast to nomothetic research, which focuses on principles that may generalize more broadly, ideographic research examines individual-, event-, and time-based idiosyncrasies [10]. EMA is characterized by (1) repeated and (2) real-time assessments in (3) naturalistic settings [1]. Phenomena of interest are measured dynamically (in contrast to statically) as they unfold (in contrast to retrospectively) and in the contexts in which they occur naturally (in contrast to the clinic, laboratory, or another location). Long-standing traditions of repeated, random sampling [11] and self-report diaries capturing naturalistic data [12] are among the bases for EMA methods.

Historically, EMA was first administered through paper-and-pencil diaries with technology available before the 21st century [1]. Currently, smartphones and tablets are the primary method for EMA delivery. Burgeoning advances have facilitated the integration of EMA with wearable devices and passive sensors, generating further naturalistic data [5]. EMA is an especially promising method for mental and behavioral health research [1]. Accordingly, EMA has been implemented across disciplines, including addiction [13], depression and anxiety [14,15], neurodevelopmental disorders [16], personality disorders [17], disruptive behavior disorders [18], eating disorders [19], schizophrenia [20], suicide [21], and chronic pain [22].

### Why Use EMA?

Self-report and other-report (eg, parent and teacher) questionnaires and laboratory-based methods are useful but limited. Questionnaires rely on retrospection, which is prone to error and may be systematically impacted by recall biases [23], and laboratory-based methods are high in internal validity but may not approximate real life [24]. Comparatively, EMA facilitates the collection of data with high ecological validity, allowing for generalization to authentic and natural individual experiences [25]. By tracing change across time relative to each individual, EMA reveals temporal associations that are inaccessible with less granular methods [24,26]. EMA has also been shown to be useful in examining dynamic clinical phenomena that are otherwise difficult to track [25,27,28].

EMA facilitates the transition from assessment to intervention. Ecological momentary interventions (EMIs), and relatedly just-in-time adaptive interventions (JITAIs), capitalize on EMA, wearable devices, and mobile device–based passive sensors to provide in-situ personalized support by detecting and adapting to individual internal and contextual states [29,30]. Identifying specific factors (eg, negative affect) that temporally precede intervention targets (eg, self-harm) via EMA can lead to novel interventions deployed in the moment that they are most needed (eg, prompting use of coping skills) via EMI or JITAI [1].

Mobile device–delivered EMA and EMI or JITAI may help increase access to mental health assessments and interventions at scale, respectively. Geographic, racial, ethnic, and socioeconomic disparities, as well as a dearth of providers, severely limit access to in-person psychological assessment and intervention [31]. Importantly, 97% of Americans report cellular phone ownership and 85% report smartphone ownership [32], which is nearly universal across gender, race, ethnicity, socioeconomic status, and aspects of psychological health [33-35]. Furthermore, internet access is expanding rapidly in low- and middle-income countries [36], providing promise for a global health impact. Accordingly, technology-facilitated assessment, intervention, and monitoring may ultimately prove to be more cost-effective and scalable than in-person alternatives [5].

### This Research

As mentioned earlier, research leveraging EMA has rapidly increased since its inception (Multimedia Appendix 1). At the time of writing, at least 63 EMA platforms were available for research use, and at least 12 research groups have developed their own platforms (Multimedia Appendix 2 [37-48]). Researchers may benefit from direct guidance on selecting an EMA platform, given the quantity of platforms available and researchers who have dedicated resources to developing their own platforms. At the National Institute of Mental Health (NIMH) and Boston Children’s Hospital (BCH), we are engaged in individual and overlapping research programs using EMA. Recognizing the unmet needs of our laboratories by our existing EMA platforms, we collaborated to select our next EMA platform.

## Methods

### Purpose and Approach

#### Team Experience With EMA Platforms

This project was the product of a collaboration between the NIMH and BCH. We represent diverse specialties in child and adolescent behavioral and mental health (irritability, chronic pain, and substance use) and a range of experiences with EMA platforms (users and developers).

##### Team at the NIMH

The focus of our research is the identification of treatment targets for psychiatric disorders and the development and evaluation of innovative, scalable, and cost-effective interventions for children and adolescents (ages 8-17 years). We have experience as users of multiple text-based and app-based EMA platforms, as well as experience as developers of “RATE-IT,” an app-based EMA platform for use in our own research studies [44]. We use EMA in isolation, as well as EMA paired with wearable devices (eg, heart rate monitors and accelerometers) [49-51]. We also have experience developing and evaluating technology-based solutions more broadly, including through assessment (eg, smartphone app–based inhibitory control assessment [52]) and intervention (ie, computer-based interpretation bias training targeting face emotion processing [53]).

##### Team at BCH

We focus on the development, validation, and implementation of mobile health solutions to assess outcomes reliably and consistently in individuals aged 7 to 24 years with chronic and acute pain, mental health, and substance use disorders. We use EMA [54,55], wearables [56,57], digital phenotyping [58], and the internet [59] to assess behavioral patterns, sleep, pain, physical mobility, motor activity, energy expenditure, and cognitive functioning. In turn, we use these constructs to evaluate and predict clinical and treatment progression, as well as to provide smartphone-based treatments. Methodologically, we have tested methods for improving adherence and retention in longitudinal mobile health studies [60].

### Procedure

**Figure 1:**
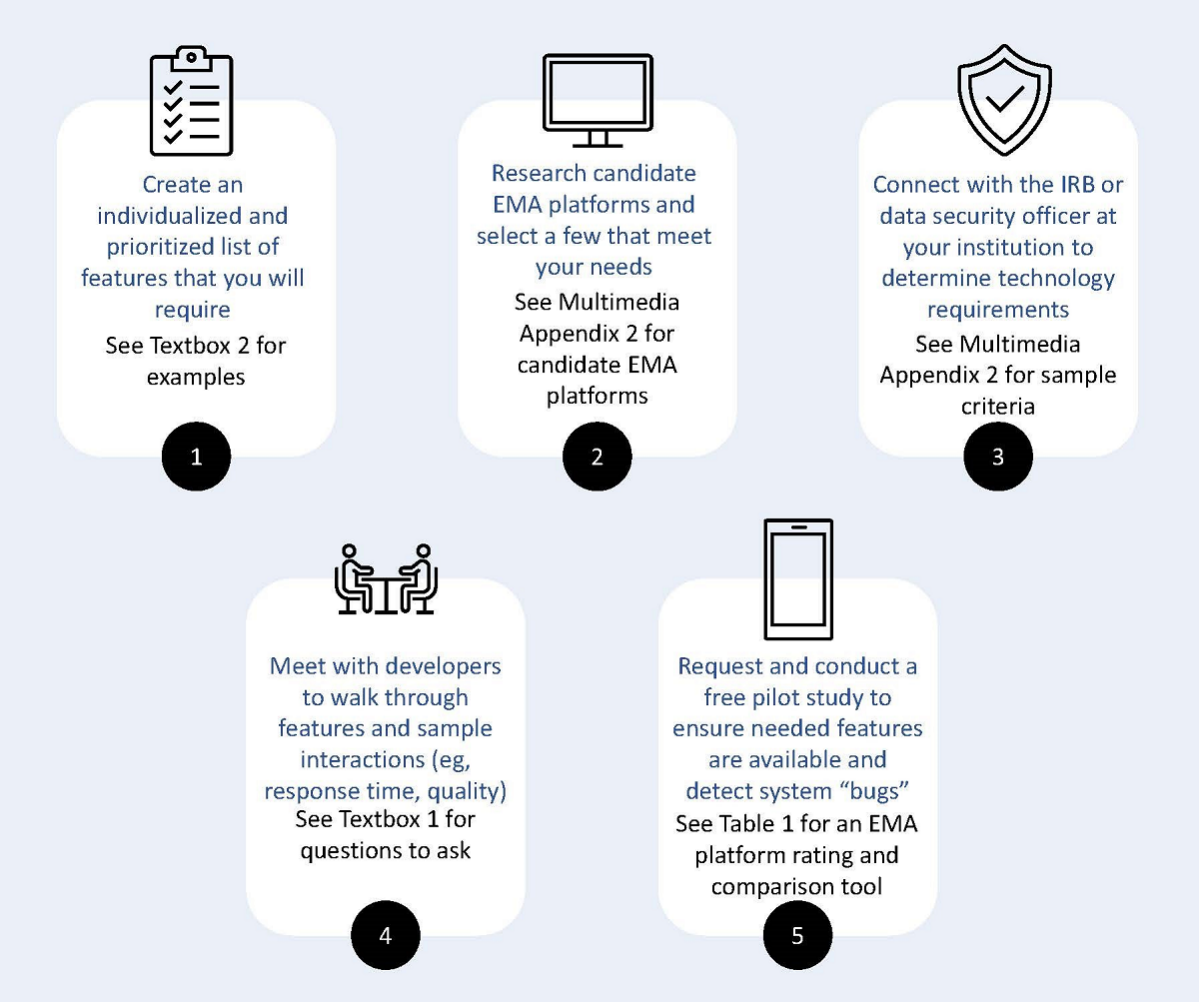
Timeline and recommendations for researchers beginning the ecological momentary assessment (EMA) platform section process. IRB: institutional review board.

We used a 5-step process to select an EMA platform (Figure 1). First, we developed a list of platform features (Textbox 1) and individualized and prioritized lists of laboratory-based needs (Textbox 2). Second, we created a list of extant platforms (Multimedia Appendix 2), conducted a web-based review to determine platform capabilities (ie, to the best of our ability, we answered questions about platform features from Textbox 1 and noted platform capabilities relative to our individualized and prioritized lists of laboratory-based needs in Textbox 2), and selected 7 EMA platforms. Third, we met with our data security officer and corresponded with our institutional review board (IRB) to determine the institutional security, privacy, and data management requirements at each of our sites (Multimedia Appendix 2). Fourth, we met with 7 platform developers to confirm our understanding of platform features; inquire about features not explicitly or clearly described on developer websites; and answer our respective institutional security, privacy, and data management questions. Fifth, we evaluated each of the 7 candidate platforms for 1 week (mean 5.00, SD 3.61 laboratory members per platform) using a combined subset of items from our laboratories.

Of note, initially, we created our list of extant EMA platforms through our team’s preexisting research-based experiences with EMA platforms; correspondence with collaborators and colleagues, including word of mouth and social media; and multiple web-based searches. For the purpose of this paper, we subsequently supplemented our initial search with data extracted by an independent rater using a search strategy implemented in a forthcoming systematic review on adherence in pediatric EMA studies (LM Henry, unpublished data, June 2023). See Multimedia Appendix 2 for the search strategy and terms.

**Ecological momentary assessment platform features and developer question examples.**

**Alarms**

- How are alarms delivered? (eg, phone calls, SMS text messages, and push notifications)
- Is the alarm design customizable? (eg, text [content, font, emphasis, color, size] and sound [type or vibration])
- Are the alarm duration and frequency programable?
- Are there options for users to delay or dismiss an alarm? Can that feature be disabled?

**Compatibility**

- With which operating systems are the platform compatible? (eg, Android and iOS)

**Consent**

- Is there an option or requirement for e-consent? Are e-consent forms fully or partially customizable?

**Cost structure**

- How is cost calculated? (eg, sample size; number of users, surveys, or items; length of study; scope of deployment [academic vs government; multisite vs single site]; extra features [integration with wearables]; type of data collected [text, audio, video]; or some combination of these factors)
- Is there an annual subscription, and how is it renewed? Are there setup fees?
- What costs are associated with developer support and consultation?
- Are discounts available? (eg, for multiyear subscriptions and student projects)

**Customization**

- Can nonessential features be removed (eg, from the menu or navigation)?
- Can you build out features that are not presently included in the platform?
- Do white label opportunities exist?

**Dashboards**

- Is a research dashboard available? If so, what are its capabilities, and is it customizable? (eg, create surveys, develop and implement a sampling scheme, message participants, track compliance, visualize data, search data)
- Is a clinician dashboard available? If so, what are its capabilities, and is it customizable? (eg, track responses, flag concerning responses, and message participants)

**Downloading results**

- When and how are user results uploaded? (eg, manually, upon Wi-Fi connection, or immediately)
- In what file format are user results downloaded?
- What information is provided in data exports? (eg, response time, missing data, compliance, and summary statistics) What structure are the data exports? (eg, long vs wide format) Is the output customizable?
- Can data be exported on a user-by-user basis? Can data be exported for the full sample?

**Dyadic interactions**

- Is there a capability for dyadic or family-level data to be linked? (eg, survey triggers based on dyadic responding or joint location)

**Gamification**

- Are there opportunities for goal setting, generic or individualized compliance feedback, rewards, badges, “streaks”?

**Item display**

- What options are available for item display? (eg, text, image, audio, video, and stopwatch)

**Item response**

- What options are available for item response? (eg, select one, select many, visual analogue, free text, image, audio, video, and geotag)

**Language capabilities**

- With which languages are the platform compatible, including infrastructure, interface, surveys, and responses?
- Can the platform automatically recognize language?

**Level of support from developers**

- Are user guides provided? (eg, written manuals and videos)
- Is developer support required or offered to facilitate setup of, and modifications to, the study?
- How are developers contacted for support? (eg, email, phone call, messaging, and website support ticket)
- Is there a direct point of contact for the study?
- In what timeframe are support requests fulfilled? (eg, in the moment, 1 business day, or 1 week)

**Messaging**

- Is there a feature that enables real-time or scheduled messaging with users during the study? (eg, general study updates, check-ins for technical difficulties)
- Are there capabilities for triggered notifications to principal investigators or clinicians for concerning responses to critical items?

**Onboarding**

- Can onboarding occur remotely or virtually, or is onboarding optimized for in-person setup?
- What method(s) are available for onboarding? (eg, email, SMS text messages, QR code, and website link)

**Passive sensing and wearable integration**

- Is wearable integration offered? If so, with which wearables can the platform pair?
- Is passive sensing offered? (eg, accelerometer, geolocation, and screen time)

**Sampling scheme and scheduling**

- What sampling schemes are supported? (eg, interval contingent, signal contingent, and event contingent)
- Can multiple forms be created?
- What are the branching logic capabilities?
- Can item responses or passive sensing trigger new surveys?
- Can surveys be deployed and completed while offline? (ie, are data and Wi-Fi required for functionality)

**Individualized and prioritized laboratory-based needs for an ecological momentary assessment (EMA) platform for teams at the National Institute of Mental Health and Boston Children’s Hospital.**

**The National Institute of Mental Health (NIMH)**

1. Meets NIMH security requirements and high likelihood of institutional review board approval
2. Child and adolescent friendly
3. Clinical and research dashboards
4. Language: compatibility with English and Hebrew
5. Self-programming option
6. Thermometer item display option and visual analog scale with midpoint item response option
7. Wearable integration
8. Passive sensing
9. Dyadic interactions

**Boston Children’s Hospital**

1. High likelihood of institutional review board approval
2. Child and adolescent friendly
3. Direct messaging with participants
4. Wearable integration
5. Research dashboard
6. Price
7. Support from developer

## Results

### Overview

Our teams at the NIMH and BCH selected 2 different platforms as the product of our evaluation. We believe that our personalized choices reflect the importance of platform selection driven by individualized and prioritized laboratory needs rather than a single platform being the ideal system for EMA researchers. Our selection processes are described in detail below. Furthermore, Table 1 depicts a 3-point rating system used by our team at the NIMH as one tool for comparing the 7 platforms on each of our individualized and prioritized needs.

**Table 1:** 3-point rating system for comparing ecological momentary assessment platforms on individualized and prioritized laboratory-based needs; example from the National Institute of Mental Health.

Individualized and prioritized need	Platform 1	Platform 2	Platform 3	Platform 4	Platform 5	Platform 6	Platform 7
Meets the National Institute of Mental Health security requirements and high likelihood of IRB^a^ approval	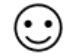 ^b^	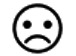 ^c^	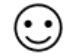	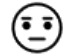 ^d^	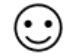	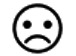	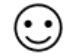
Child and adolescent friendly	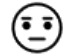	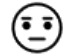	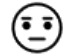	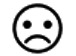	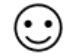	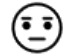	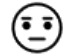
Clinical and research dashboards	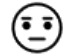	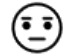	?^e^	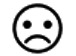	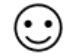	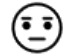	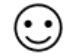
Language: compatibility with English and Hebrew	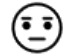	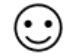	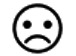	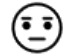	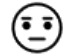	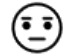	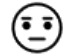
Self-programming option	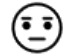	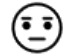	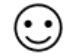	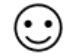	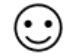	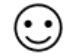	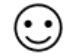
Thermometer item display option and visual analog scale with midpoint item response option	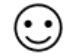	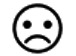	?	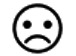	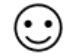	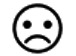	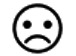
Wearable integration	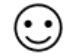	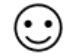	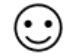	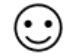	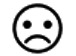	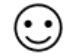	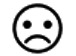
Passive sensing	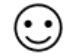	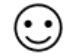	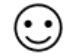	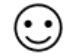	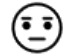	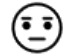	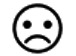
Dyadic interactions	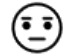	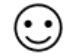	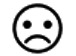	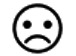	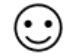	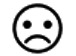	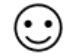

^a^IRB: institutional review board.

^b^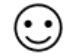: full capability.

^c^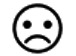: no capability.

^d^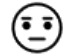: some capability or requires modifications to achieve full capability.

^e^?: no information.

Our team at the NIMH ultimately selected platform 5.

### Platform Selection

Our teams selected platforms that were, for the most part, comprehensive in standard features (eg, alarms, device compatibility, downloading results, item display and response, onboarding, sampling schemes, and scheduling; Textbox 1). Unavailable features were anticipated to be added within the next year (ie, audio item response) or could be added for an additional cost (ie, image and video item response) and importantly were not necessary for our ongoing or upcoming laboratory studies.

A subset of our individualized and prioritized laboratory-based needs (Textbox 2) were shared across teams at the NIMH and BCH. Compliance with institutional data security requirements and a high likelihood of IRB approval were paramount (priority 1). Through meetings and correspondence with developers, we obtained answers to the relevant data security questions. At the NIMH, our selected platform did not initially meet security requirements because of data storage outside the United States. However, the developers proposed a viable technical solution for an additional cost. In addition, both teams valued child- and adolescent-friendly user interfaces (priority 2). Our team at BCH recruits a wider age range (7-24 years) than our team at the NIMH (8-17 years). Therefore, BCH selected a platform with customizable fonts, colors, and graphics that would allow for tailoring, depending on the study and sample. The NIMH selected a platform with a fixed, simple, and brightly colored user interface, child-friendly item displays (eg, smiley faces), and gamified incentives. Although both teams were interested in wearable integration, we prioritized this feature differently (priority 4 at BCH and priority 7 at the NIMH). At BCH, a majority of our studies use actigraphy devices to collect activity and sleep data. Thus, the seamless integration of EMA and wearable device data was highly valued. At the NIMH, no ongoing studies actively integrate EMA and wearable devices. In fact, wearable integration was the only item on the NIMH’s list of individualized and prioritized laboratory-based needs not offered by our selected platform. Although several other platforms offered wearable integration, we decided to forgo it given the low prioritization. In addition, both teams valued research dashboards that would facilitate the tracking of individual- and sample-level EMA survey compliance (priority 3 at the NIMH; priority 5 at BCH). At the NIMH, we additionally required a dashboard for clinician use (eg, visualizing participant responses to critical clinical items, priority 3). The platforms that we selected conformed to our clinical and research dashboard needs. Finally, we considered interactions with developers, including response time and quality. Both teams had troubleshot countless technical errors on researcher and participant platform interfaces in previous research. At BCH, it was important for our team to select a platform with a strong developer support system (priority 7). Although not originally prioritized at the NIMH, developer interactions contributed to our decision-making. Across teams, the developers of our selected platforms were quick and comprehensive in correspondence and were willing to collaborate with our teams to address concerns as they developed. In contrast, for example, developers from one platform did not respond to our email inquiries regarding their features (Table 1).

A subset of individualized and prioritized laboratory-based needs were unique to BCH. EMA adherence was a challenge in our previous study. Therefore, we prioritized direct messaging capabilities to send participants survey reminders, troubleshoot issues with wearable devices, and inquire about adherence (priority 3). Platform pricing was also a consideration (priority 6). We compared platform pricing and packages (eg, the maximum number of participants and studies allowed) relative to our needs and the corresponding platform features.

A subset of individualized and prioritized laboratory-based needs were unique to the NIMH. All evaluated platforms were fully or partially (eg, item and response options but not app infrastructure, such as menu options) compatible with Hebrew with additional customization (priority 4). Five platforms, including our selected platform, were entirely self-programmable (priority 5); 2 platforms required partnering with developers for the study setup and allowed subsequent self-programmed modifications. Our selected platform did not initially offer our desired thermometer item display or visual analog scale with midpoint item response options (priority 6), but the developers built these options free of charge in time for our evaluation. Platforms ranged in passive sensing capabilities (priority 8), with some offering full, continuous passive sensing for multiple metrics (eg, text transcripts, step count, browser history, and social media posts); others offering passive sensing during (or close in time to) survey completion (our selected platform); and still others without passive sensing capabilities. Finally, our selected platform was 1 of 3 evaluated platforms with dyadic interaction capabilities (eg, triggering surveys based on the responses of a designated partner; priority 9).

## Discussion

### Overview

On the basis of our experiences as EMA platform users and developers, along with lessons learned from the current research, here, we discuss 11 considerations in selecting an EMA platform: (1) location; (2) developer involvement; (3) sample characteristics; (4) onboarding; (5) survey design features; (6) sampling scheme and scheduling; (7) viewing results; (8) dashboards; (9) security, privacy, and data management; (10) pricing and cost structure; and (11) future directions. For each consideration, we provide example questions to ask platform developers, which vary based on individualized and prioritized lab-based needs (Textbox 1).

### Consideration 1: Location

#### Overview

Think about the location of the developer relative to your study and institution. The developer’s location may influence the language or languages of the participant and researcher platform interfaces and instructional materials. In addition, developer location may impact the timing of support from developers and dictate the location of the data storage.

#### Are Platform Features and Instructions Available in (My Language or Languages)?

Determine whether each platform is available in the language or languages of your sample and study team. It is not only necessary to confirm that survey items and response options are programmable in the language or languages of your study, but it is also important that participant- and researcher-facing features are available in the appropriate language or languages. Can the platform accommodate languages with non-Roman alphabets (eg, Arabic and Greek) or languages that read from right to left (eg, Hebrew and Farsi)? Some developers are willing to build new languages into their platforms (potentially for a fee). Furthermore, developers may offer knowledge bases, manuals, or other informational materials that will help you use the platform, and it is important that these resources are accessible to your team.

#### Will You Be in Office While (My Study) Is Live, so That I Can Receive Support, if Needed?

If developer support with quick turnaround time is a priority, determine when your study will be live, when developers will be available, and how quickly developers will respond to service requests. Consider the time zone differences between the developer and study sites. Additional coordination may be necessary if there are gaps in developer coverage. For example, in previous research, our team at BCH scheduled a weekly check-in and troubleshooting meeting with developers outside the United States to circumvent response delays due to time zone differences.

#### Where Will the Data Be Stored?

Determine whether your country and institution impose restrictions on the location of your data. For example, the General Data Protection Regulation stipulates how the data of European Union citizens and residents must be collected, stored, processed, and disseminated (eg, individuals must have the ability to erase their personal data), and platforms that do not comply may not be used [61]. Regarding institutional policies on data storage, early conversations with your data security officer or IRB are critical (see the section *Consideration 9: Security, Privacy, and Data Management*). If data storage outside of your country is prohibited by your institution, some developers may collaborate on customizing data storage locations.

### Consideration 2: Developer Involvement

#### Overview

Although some studies are straightforward and require low maintenance, others are more complex and require more frequent communication with developers. Relative to the technical skill levels of your team members, consider the support (type and quantity) you will need from developers at the beginning and throughout the life span of your study.

#### How Much Support Will You Provide at the Beginning of (My Study)?

Developers offer varying levels of support when initiating a new study, and accordingly, our teams encountered a range of support at the outset of our evaluation. Some developers programmed the study on our behalf, in part or in whole, after receiving our protocol. Other developers provided access to knowledge bases, manuals, and other informational materials to facilitate the setup led by our teams. The cost of developer involvement in the study setup varies. Some platforms offer unlimited support or build a fixed number of training sessions into their pricing. Other platforms support users on a fee-per-session basis.

#### How Much Support Will You Provide Throughout the Life Span of (My Study)?

Developers offer varying levels of support throughout the life span of a study. In our evaluation, developer response times spanned hours to weeks. Typically, we requested support by emailing a prespecified point of contact or submitting “help tickets” on the web. If support is provided via email, work with developers to determine an alternate point of contact for vacation and sick days. If support is provided via help tickets, inquire about the average time from receipt to resolution. Regardless, be informed and develop a backup plan. Plan ahead, as most technical malfunctions tend to occur when studies are launched.

### Consideration 3: Sample Characteristics

#### Overview

The characteristics of your sample may inform the optimal participant user experience (UX) for your platform.

#### Is Your Participant UX Optimized for (My Sample Characteristics)?

Intuitive and engaging UX is a goal for developers, regardless of sample characteristics. Still, depending on factors, including participant age range and level of cognitive functioning, it may be critical to implement an easy-to-navigate and visually appealing UX. For example, the NIMH prioritized a child-friendly UX. Of the platforms we evaluated, a few were characterized by child-friendly UX (eg, large fonts and bright colors), and some (but not all) allowed for user customization. Gamification and rewards may increase survey compliance and minimize dropout. Of the platforms that we evaluated, one offered awards for survey completion and another offered a virtual “shop” to unlock incentives such as fun facts. Creative response options may make items clearer and more engaging. In our evaluation, one platform offered emoji response options and another platform enabled researchers to upload images as response options.

#### What Features Support Platform Use With High-Risk Samples?

If recruiting a high-risk sample (eg, individuals at risk for suicidal ideation), you may consider platforms that allow for responses (eg, thoughts of death or dying) to be “flagged” and forwarded to a point of contact on your team (eg, clinician) for follow-up. Some platforms leverage natural language processing to identify flagged terms in free-response items. In addition, some platforms can be programmed to automatically generate follow-up questions or messages for a flagged response.

### Consideration 4: Onboarding

#### Overview

Determine whether you will enroll participants in person or virtually, factoring in the technical skills of your team members and sample, and assess the relative fit of platform onboarding options.

#### How Can Participants Enroll in (My Study)?

Consider how participants will enroll in your study. Although participants may benefit from in vivo technical support, virtual onboarding may be less resource-intensive (eg, lower cost, easier to schedule). Both the technical skills of your sample (considering age, cognitive functioning, and clinical status) and research team are factors to consider. Some platforms autogenerate onboarding invitations that are sent to participants via email or SMS text message. Notably, email invitations may be sent to spam folders, emphasizing the importance of detailed study protocols, including guidance on troubleshooting technical issues during onboarding. Other platforms direct participants to scan a QR code for study enrolment. The level of automaticity and researcher time and effort in onboarding varies by platform. For example, when evaluating one platform, our team was responsible for generating mobile codes for enrollment.

#### Can or Must Participants Provide Consent Through the Platform?

Some platforms allow for the administration of a researcher-developed e-consent form as part of onboarding. Understand whether this feature is available if needed and can be disabled if you decide to consent participants through another method. In addition, given passive sensing integrations to some platforms, participants may be prompted to provide permission to access smartphone features and sensors (eg, microphones). In our evaluation, multiple participants expressed concerns about privacy upon receiving smartphone sensor permission requests from the platform, even though we instructed participants to refuse those requests and did not collect passive data.

### Consideration 5: Survey Design

#### Overview

Determine which design features are required for your surveys to be maximally effective, user-friendly, and tailored to your sample.

#### Does the Platform Offer (My Prioritized Survey Display Options)?

Determine whether you require your surveys to display text, images, audio, or video to the user. Depending on your sample, visual or auditory cues may be beneficial (see the section *Consideration 3: Sample Characteristics*). Although text display is ubiquitous across platforms, options to present images, audio, and video vary by platform. In addition, consider how you would like users to move through your survey; some platforms display multiple items on a single page (ie, via scrolling), other platforms display one item per page, and still others allow for customization. Think about whether participants should be able to skip items and return to previous items and determine if those features are modifiable in the platform.

#### Does the Platform Offer (My Prioritized Survey Response Options)?

Determine whether your response options will be a single-choice, multiple-choice, Likert-scale, or free-text response or something else. For example, you might ask participants to respond through captured images, audio, video, time stamps, or geotags. In addition, consider your need for “branching logic” or custom paths based on user responses. Many platforms allow participant responses to dictate the items to follow, but fewer platforms allow responses to trigger future surveys (eg, later in the day).

### Consideration 6: Sampling Scheme and Scheduling

#### Overview

Determine the sampling scheme for your study and whether the platform supports your sampling scheme. Consider the other platform features you may need for successful survey deployment and completion.

#### Which Sampling Schemes Are Supported by the Platform?

Decide whether your surveys will be interval contingent, signal contingent, event contingent, or some combination thereof. Some platforms also allow for pairing with wearable devices and passive sensors, such that geolocation or another target might trigger a survey. Inquire as to whether multiple surveys can be programmed and deployed or if the platform can only manage a single survey. In addition, understand the method for developing the sampling scheme structure within the platform; our team found the evaluation to be critical in testing the researcher-facing platform design.

#### What Aspects of the Alarm or Notification System Are Modifiable?

Alarms signaling users to complete a survey might use sound, vibration, a banner, or a push notification. Some platforms allow for reminder notifications if a survey is not completed. Understand the level of customization offered by your platform. Greater flexibility will allow you to develop a system tailored to your sample such that participants remain engaged but not overwhelmed with notifications.

#### Can Survey Scheduling Vary Based on Participant Personal Preferences, or Is Scheduling Fixed Across Participants?

Some degree of personalization in survey scheduling may increase survey compliance. For example, the morning survey for participant A (an early riser) may be deployed randomly between 7 AM and 8 AM, and the morning survey for participant B (a late sleeper) may be deployed randomly between 8:30 AM and 9:30 AM. Determine whether the platform allows for personalization or if survey scheduling must be held constant across your sample. In addition, when enrolling participants across time zones (eg, multisite studies), understand whether the survey will be deployed at the local time of the participant or researcher.

#### Does the Platform Require Wi-Fi or Cellular Data to Function?

Understand the impact of participant phones being “offline” (eg, survey deployment, response, and upload to the server). For example, some platforms are native apps that allow users to access and respond to surveys even offline, and other platforms store responses locally until Wi-Fi or cellular data are available. In addition, consider the impact of the platform on data use. Significant data use may be prohibitive if participants use their own phones. Passive sensing may rely heavily on data.

### Consideration 7: Dashboards

#### Overview

Dashboards can be used for data monitoring. Given that dashboards are not universal to platforms, consider whether a dashboard is a priority.

#### Do You Offer Dashboards, What Are Their Capabilities, and to What Extent Are They Customizable?

Dashboards can be programmed to report high-level information about your sample, specific information about individual participants, or both. When determining your dashboard needs, consider which team members will need to view your data (eg, research coordinator, clinician, or principal investigator). Then, consider which variables each team member will need to view (eg, compliance and symptoms), including level (eg, full sample and individual participants) and interval (eg, daily and weekly). In addition, consider how the data should be visualized (eg, graphs and tables). For example, clinicians may want to track depression symptom scores weekly over the course of a clinical trial using a time-series graph. Some groups may be interested in data collection on dyads, making platforms with dyadic linking and dashboards with side-by-side data visualization optimal. If multiple users require dashboards for distinct purposes (eg, clinicians and researchers), inquire whether the platform supports multiple dashboards and users in a single study.

### Consideration 8: Viewing Results

#### Overview

Consider how and how often data should be uploaded to the server and downloaded by your research team. In addition, consider your preferences for structuring your data export.

#### How and How Often Are Data Uploaded?

Consider how (eg, Wi-Fi and cellular data) and how often (eg, immediately and daily) data are uploaded to the server. Some platforms require Wi-Fi or cellular data to deliver surveys and subsequently upload data. Other platforms are accessible offline and do not require connections to Wi-Fi or cellular data for functionality. Still other platforms deliver surveys offline, and data are stored on the platform and uploaded when connected to Wi-Fi or cellular data. Data upload considerations may vary in importance depending on your study design (eg, use of participant phones compared with study phones).

#### In What Formats Are Data Exported?

In many cases, platforms export data as comma-separated values files. Some platforms offer additional formats (eg, JavaScript Object Notation) and application programming interfaces (eg, SPSS and R). In addition, some platforms export individual participant data (which may be less feasible for data collection on large samples) and others export data for the full sample.

#### How Will the Data Export Be Structured?

Platforms vary in how their data exports are structured and whether (and to what extent) their data exports are customizable. Consider whether you will need your data in a long or wide format, how missing data should be reported, and which fields are necessary (eg, skipped items, response time, and summary statistics). Inconsistency between data exports and your needs will yield extra time and effort in preprocessing.

### Consideration 9: Security, Privacy, and Data Management

#### Overview

Consider security, privacy, and data management requirements as they relate to your country and institution. The presence or absence of certain platform features (eg, 2-factor authentication; 2FA) may render a platform incompatible with those requirements. Depending on your institution and familiarity with their policies, consider meeting or corresponding with your data security officer or IRB before consulting with developers. Our team at the NIMH met with our data security officer to understand the requirements of IT applications and services (Multimedia Appendix 2). We presented the corresponding questions to developers when we met to discuss their platforms. On the basis of developer responses, our data security officer assigned platforms to varying levels of security clearance for NIMH-specific standards, which informed the selection of our platform. Our team at BCH was not required to have an initial conversation with data security. Instead, our IRB application included questions about our candidate platform (Multimedia Appendix 2), and we met with the data security team following submission.

#### How Are the Platform and Data Secured?

Inquire about whether and how the platform secures access, including requiring a personal identification number, a password, or 2FA. Although 2FA by some means (eg, email) may complicate, slow, and impede survey responses, other means may be simpler, quicker, and more feasible (eg, Touch ID, Face ID, and text). Some of the platforms we evaluated offered (and some required) 2FA. Other platforms did not offer 2FA, stating that study accounts do not store user phone numbers to enable 2FA. In addition, inquire about whether and how data are stored on the platform and mobile device and how data are encrypted. All the platforms evaluated encrypted data as they were transported between phones and servers, and most platforms used Amazon Web Services for cloud storage.

#### How Does the Platform Maintain User Privacy?

Ask developers what kind of information is stored about the participant (eg, log-in information, survey data, and location), where (eg, on the platform and in a server), and for how long (eg, days, months, duration of the contract, or in perpetuity). Follow up to determine how data are deidentified, if at all, and where the key to the data is stored. Inquire as to whether the platform is compliant with the laws and regulations of your country that protect an individual’s data or information (eg, Health Insurance Portability and Accountability Act in the United States and General Data Protection Regulation in the European Union). In our evaluation, platforms typically temporarily store survey data on phones in an encrypted format not visible in phone storage or accessible to other apps. These data were deleted from the phone once they were uploaded to the server. Some platforms avoid storing user information by not creating user accounts. Other platforms create user accounts but do not require personal information. Still others use information such as email addresses to create accounts.

#### How Are Data Managed?

Determine whether the data will be stored in the country of research or abroad (see the section *Consideration 1: Location*) and on a physical server or the cloud. Determine how long the platform will retain the data, along with whether and when long-term data storage will incur an additional cost. During our evaluation, one platform maintained data storage for the license period and another platform maintained data storage for 6 months following the license period to allow researchers time for data analysis.

### Consideration 10: Pricing and Cost Structure

#### Overview

Before meeting with developers, know your budget. The platforms we evaluated offered complimentary project consultations and estimates, which were key in selecting among candidates.

#### What Is Your Pricing and Cost Structure?

Platforms vary widely in their pricing and cost structures. Platforms may charge a flat fee for a user license with varying levels (basic and premium), or platforms may provide quotes that are team and project specific, varying depending on your estimated number of participants, the size of your team, the roles of your team members (eg, researchers and clinicians), your institution, and required features (eg, basic EMA vs EMA plus passive sensing). Some platforms offer free, basic versions of their platform. For example, one platform in our evaluation offered a basic package for clinicians to use in private practice with their clients while researchers were asked to purchase a premium package. Another platform offered discounted costs with the purchase of a multiple-year license. Costs may also vary based on whether you are part of an academic institution or a government institution and whether your project is single or multisite. Academic institutions are typically lower in cost than private entities, and single-site projects are lower in cost than multisite projects.

#### What Additional Features Do You Offer and Are They Included in the Price?

Consider how necessary, desired, and unnecessary features may impact platform costs. Sophisticated tools such as passive sensing, pairing with wearable devices, dyadic linking of participants, or compatibility with health care systems are costly to develop and maintain. Furthermore, images and videos may be expensive for developers to store. One of the platforms that we evaluated offered advanced features, including the ability to access electronic health records and facilitate biospecimen collection, which resulted in high overall costs for the platform. If your project requires only EMA, choose a platform with basic offerings or ask if you can opt out of extra features.

### Consideration 11: Future Directions of Your Study and Research Program

#### Overview

The next steps for the platform (eg, financial viability and developments) and your study and research program (eg, the need for additional features) should be considered before you partner with developers.

#### How Long Do You Expect the Platform to Be Operational?

Consider the length of your study and explicitly ask developers their expectation for being operational for that duration and longer. A single, reliable platform is critical for stable data collection. Funding sources may inform the long-term stability of a platform. For example, if a platform is grant funded, inquire about plans for financing the platform when the funding period elapses. In addition, ask whether and when developers plan to launch upgrades to, or new versions of, the platform and how those developments might interface with an ongoing study. These considerations are especially critical for longitudinal studies.

#### How Could the Platform Adapt to the Evolving Needs of (My Laboratory)?

As you plan your research program (eg, integration of additional measures delivered via EMA, passive sensing, and wearable devices), consider whether the platform has or can develop the capacity to support these plans. At the same time, it may be appropriate to select a particular platform for one phase of research with the understanding that the platform may be reevaluated in the future.

### Conclusions

EMA is a tool for the repeated, real-time assessment of constructs of interest in natural settings, which offers great opportunity for advancing psychological science. Accordingly, interest in incorporating EMA into individual studies and research programs has burgeoned since EMA emerged in the 1990s. However, numerous platforms are available to researchers, and choosing between options is an arduous task to the point that some researchers elect to develop their own platforms. Taken together, we found that the optimal EMA platform is the one that best meets the study and laboratory needs. Reconciling needs with opportunities to optimize outcomes requires information, understanding, and planning. Here, we offer specific guidance on platform selection for researchers interested in integrating EMA methods into their research programs.

## Appendix

Multimedia Appendix 1 Number of publications by year from 1994 through 2023 including the term ecological momentary assessment (EMA) and related terms. Overall, 1032 publications in 2012 and 2767 publications in 2022 used the term EMA and related terms, yielding an increase of over 168% across time. These data reflect a search of Embase, PsycINFO, Web of Science, Scopus, and PubMed from 1994 (when the term EMA was coined by Stone and Shiffman) through 2023, which yielded 25,353 articles. An additional 1061 articles were published from 1829 through 1993 using the term “daily diary,” a technique preceding EMA consisting of a single end-of-day assessment typically administered via paper and pencil or a telephone call.

Multimedia Appendix 2 A list of app-based, text-based, web-based, and user-design ecological momentary assessment (EMA) platforms; a list of EMA platforms developed by researchers for individual use; the PubMed search strategy; and sample EMA platform data security questions.

## Abbreviations

2FA: 2-factor authentication
BCH: Boston Children’s Hospital
EMA: ecological momentary assessment
EMI: ecological momentary intervention
IRB: institutional review board
JITAI: just-in-time adaptive intervention
NIMH: National Institute of Mental Health
UX: user experience
