# Supplementary figures and images for: Selecting an Ecological Momentary Assessment Platform: Tutorial for Researchers
Source: J Med Internet Res. 2024 Jan 4;26:e51125. doi: 10.2196/51125 (PMC10797510; doi:10.2196/51125)

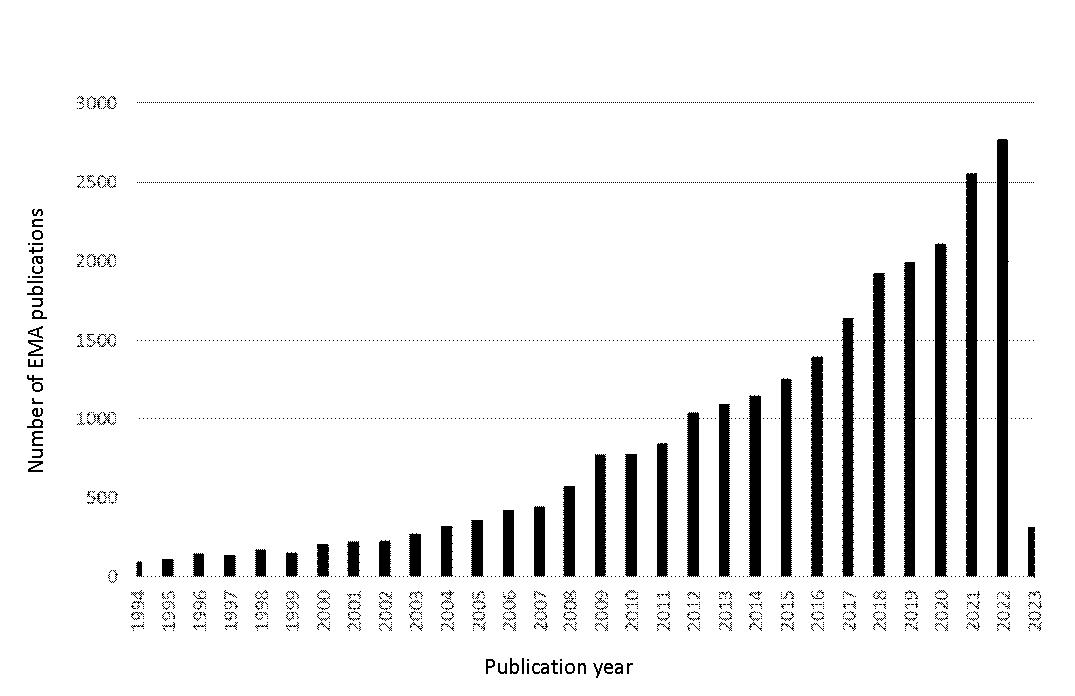

Supplement: Multimedia Appendix 1 [file jmir_v26i1e51125_app1.png]
