# Supplementary material for: Selecting an Ecological Momentary Assessment Platform: Tutorial for Researchers
Source: J Med Internet Res. 2024 Jan 4;26:e51125. doi: 10.2196/51125 (PMC10797510; doi:10.2196/51125)
Supplement: Multimedia Appendix 2 [file jmir_v26i1e51125_app2.docx]

Multimedia Appendix 2

**Table S1**. App-based, text-based, web-based, and user design ecological momentary assessment (EMA) platforms^a^.

| Name | | Developer^b^ (country) | | Website | | | Passive sensing features, wearable integration, and EMI^c^ capabilities |
| --- | --- | --- | --- | --- | --- | --- | --- |
| **App-based platforms** | | | | | | | |
| AthenaCX | | AthenaCX  (Ireland) | | https://www.athenacx.com/ | | | - Passive sensing: geolocation - Wearables: pairs with Fitbit (eg, activity, physiology) - EMI: NR^d^ |
| AWARE-Light | | AWARE (Australia) | | https://www.aware-light.org/ | | | - Passive sensing: accelerometer, app use, barometer, battery, Bluetooth, calendar, geolocation, gravity, gyroscope, installations, keyboard, light, magnetometer, Message Queuing Telemetry Transport, network, phone processor, phone proximity, phone rotation, screen time, telephony, temperature, text communications, text2speech, time zone, Wi-Fi^e^ - Wearables: NR - EMI: NR |
| Beiwe | | Harvard University, Dr Jukka-Pekka Onnela  (United States) | | https://beiwe.wpengine.com/ | | | - Passive sensing: accelerometer, accuracy column, Bluetooth, DeviceMotion, geolocation, gyroscope, identifiers, magnetometer, phone call log, power state, proximity, reachability, screen time and phone use, SMS text message log, Wi-Fi router^e^ - Wearables: NR - EMI: NR |
| ClinTrak | | MedPace  (United States) | | https://www.medpace.com/about/trusted-by-biotech/ | | | - Passive sensing: NR - Wearables: NR - EMI: NR |
| Colliga | | | Colliga Apps  (United States) | | https://colliga.io/ | - Passive sensing: accelerometer, ambient noise, call frequency and duration, email browser history, geolocation, heart rate, humidity, proximity to other users and time spent together, sleep duration, social media and SMS transcripts, social media posts, step count - Wearables: NR - EMI: yes | |
| eCOA Multimedia | | | Clario  (United States) | | https://clario.com/solutions/ecoa/ecoa-multimedia/ | - Passive sensing: NR - Wearables: integrates with wearables for mobility measures (eg, sit to stand postural transition) - EMI: NR | |
| eMoodie | | | eMoodie  (Scotland) | | https://emoodie.com | - Passive sensing: accelerometer, pedometer, gyroscope, time on voice calls, number of incoming and outgoing text messages, app use, ambient light, temperature - Wearables: NR - EMI: yes | |
| Enketo | | | Enketo LLC  (United States) | | https://enketo.org/ | - Passive sensing: NR - Wearables: NR - EMI: NR | |
| EpiCollect5 | | | Centre for Genomic Pathogen Surveillance  (United Kingdom) | | https://five.epicollect.net/ | - Passive sensing: NR - Wearables: NR - EMI: NR | |
| Esm Capture | | | EsmCapture  (NR) | | https://esmcapture.com/ | - Passive sensing: geolocation - Wearables: NR - EMI: NR | |
| ESMira | | | University of Health Services  (Austria) | | https://esmira.kl.ac.at/?about&lang=en | - Passive sensing: NR - Wearables: NR - EMI: NR | |
| Ethica | | | Ethica Data  (Canada) | | https://ethicadata.com/ | - Passive sensing: accelerometer, activity type, app use, call and text contacts, geolocation, proximity to Bluetooth devices, screen time, step count - Wearables: pairs with Garmin, Apple Watch, Fitbit, and Google Fit - EMI: yes | |
| ExperienceSampler | | | ExperienceSampler  (Canada) | | http://www.experiencesampler.com/index.html | - Passive sensing: NR - Wearables: NR - EMI: NR | |
| Expiwell | | | Expiwell  (United States) | | https://www.expiwell.com/ | - Passive sensing: accelerometer, geolocation - Wearables: NR - EMI: yes | |
| formr | | | Georg August University Göttingen, Friedrich Schiller University Jena, Ruben Arslan and Cyril Tata  (Germany) | | https://formr.org/ | - Passive sensing: NR - Wearables: NR - EMI: NR | |
| Inquisit | | | Millisecond Software LLC  (United States) | | https://www.millisecond.com/ | - Passive sensing: NR - Wearables: NR - EMI: NR | |
| iSurvey | | | HarvestYourData  (United States) | | https://www.harvestyourdata.com/ | - Passive sensing: geolocation - Wearables: NR - EMI: NR | |
| mEMA | | | Ilumivu  (United States) | | https://ilumivu.com/ | - Passive sensing: accelerometer, ambient light and sound, geolocation, phone use, weather - Wearables: pairs with Garmin, Empatica, and Apple Watch (eg, heart rate, heart rate variability, steps, sleep, and blood pressure) - EMI: yes | |
| MetricWire | | | MetricWire Inc  (Canada) | | https://metricwire.com/ | - Passive sensing: altitude, geolocation, motion type, steps, and walking and running speed - Wearables: pairs with Fitbit and Garmin - EMI: yes | |
| mindLAMP | | | LAMP Consortium and Division of Digital Psychiatry  (United States) | | https://docs.lamp.digital/ | - Passive sensing: analytics, accelerometer, activity recognition, Bluetooth and Wi-Fi, blood glucose, blood pressure, body temperature, calls and texts, device motion, geolocation, heart rate, heart rate variability, nutrition, oxygen saturation, pedometer, pairs with wearables to track, respiratory rate, screen time, sleep, and workouts - Wearables: NR - EMI: yes | |
| MindLogger | | | Child Mind Institute  (United States) | | https://mindlogger.org/ | - Passive Sensing: NA^f^ (geolocation via a button the participant presses) - Wearables: NR - EMI: yes | |
| MindSampler | | | MindSamper (Spain) | | https://www.mindsampler.com/ | - Passive Sensing: geolocation, step count, noise levels, and phone battery - Wearables: NR - EMI: NR | |
| movisensXS | | | movisenXS  (Germany) | | https://www.movisens.com/en/ | - Passive sensing: accelerometer, geolocation, and sleep monitoring - Wearables: pairs with developer-designed wearables for activity levels, electrocardiogram, electrodermal activity, metabolic equivalent of task, and step count - EMI: yes | |
| m-Path | | | KU Leuven, Drs Merijn Mestdagh and Stijn Verdonck  (Belgium) | | https://m-path.io/landing/ | - Passive Sensing: accelerometer, activity, ambient noise, app use, battery, Bluetooth devices, device type, geolocation, light sensor, pedometer, screentime, weather, Wi-Fi connectivity - Wearables: NR - EMI: yes | |
| MuPsych | | | University of Jyväskylä, Dr Will Randall  (Finland) | | https://www.mupsych.com/ | - Passive Sensing: Music application use - Wearables: NR - EMI: NR | |
| MyCap | | | REDCap  (United States) | | https://projectmycap.org/ | - Passive sensing: NR (offers “active tasks” via Apple sensors) - Wearables: NR - EMI: NR | |
| MyDataHelps | | | Care Evolution  (United States) | | https://careevolution.com/mydatahelps/ | - Passive sensing: accelerometer, air quality, ambient light, device configuration and use, geolocation, health information, heart rate, nutrition and weight, and phone use - Wearables: Fitbit, Apple, Android, blood pressure cuff, and glucometer - EMI: yes | |
| NeuroUX | | | NeuroUX Inc  (United States) | | https://www.getneuroux.com/ | - Passive sensing: NR - Wearables: Fitbit and Google Fit (eg, physical activity, heart rate, and sleep) - EMI: NR | |
| ODK | | | Get ODK Inc  (United States) | | https://getodk.org/ | - Passive sensing: geolocation - Wearables: NR - EMI: NR | |
| ohmage | | | University of California, Los Angeles Institute for Research on Labor and Employment  (United States) | | https://engagedresearch.wixsite.com/engagedresearch/ohmage | - Passive sensing: available but features not specified - Wearables: NR - EMI: yes | |
| Paco | | | Paco  (United States) | | https://pacoapp.com/ | - Passive sensing: available but features not specified - Wearables: NR - EMI: NR | |
| Pathverse | | | Pathverse Inc  (Canada) | | https://pathverse.ca/en/ | - Passive sensing: NR - Wearables: integrates with Apple Watch and Garmin (eg, heart rate and fitness data) - EMI: yes | |
| Pendragon Forms | | | Pendragon Forms  (United States) | | https://www.pendragonforms.com/index.html | - Passive sensing: geolocation - Wearables: NR - EMI: NR | |
| PIEL Survey | | | Blue Jay Ventures  (United States) | | https://pielsurvey.org/ | - Passive sensing: NR - Wearables: NR - EMI: NR | |
| PiLR | | | PiLR Health  (United States) | | https://pilrhealth.com/ | - Passive sensing: geolocation - Wearables: NR - EMI: yes | |
| PsyMate | | | Maastricht University, SmarteHealth, Health Foundation Limburg  (Netherlands) | | https://www.psymate.eu/ | - Passive sensing: activity, geolocation - Wearables: NR - EMI: NR | |
| RealLife Exp | | | LifeData  (United States) | | https://www.lifedatacorp.com/ | - Passive sensing: geolocation - Wearables: NR - EMI: NR | |
| Samply Research | | | Samply Research, University of Konstanz, Dr Yury Shevchenko  (Germany) | | https://samply.uni-konstanz.de/ | - Passive sensing: NR - Wearables: NR - EMI: NR | |
| Scene | | | Scene Health  (United States) | | https://www.scene.health/ | - Passive sensing: NR - Wearables: NR - EMI: yes | |
| SEMA3 | | | Melbourne eResearch Group  (Australia) | | https://sema3.com/ | - Passive sensing: NR - Wearables: NR - EMI: NR | |
| Sensus | | | University of Virginia  (United States) | | https://cacs.virginia.edu/our-work/sensus-mobile-app | - Passive sensing: geolocation, pedometer, physical activity, sleep, and other health measures - Wearables: NR - EMI: NR | |
| SleepLife | | | Dogtown Media, LLC  (United States) | | https://www.dogtownmedia.com/portfolio/sleeplife/ | - Passive sensing: NR - Wearables: pairs with Fitbit (eg, total sleep time, sleep efficiency, and awakenings) - EMI: NR | |
| Symptom Tracker | | | Gastric Imaging & Analysis GmbH  (Switzerland) | | https://www.gastric.ch/symptom-tracker/ | - Passive sensing: NR - Wearables: NR - EMI: NR | |
| Teamscope | | | Teamscope  (Netherlands) | | https://www.teamscopeapp.com/ | - Passive sensing: NR - Wearables: NR - EMI: NR | |
| TigerAware | | | TigerAware  (United States) | | https://tigeraware.com/ | - Passive sensing: accelerometer, geolocation - Wearables: pairs with wearables - EMI: NR | |
| TrackYourTinnitus | | | Tinnitus Research Initiative, Ulm University  (Germany) | | https://www.trackyourtinnitus.org/home | - Passive sensing: NR - Wearables: NR - EMI: NR | |
| WellMind | | | NEATLabs, UC San Diego, Drs Jyoti Mishra and Dhakshin Ramanathan  (United States) | | https://neatlabs.ucsd.edu/ | - Passive sensing: contact developers for information - Wearables: contact developers for information - EMI: yes | |
| Youcognize | | | Mentegram  (United States) | | http://youcognize-web.beta-webs.com/what-is-youcognize/app/ | - Passive sensing: NR - Wearables: NR - EMI: NR | |
| **Text- or web-based platforms** | | | | | | | |
| Forsta | | | Forsta  (United Kingdom) | | https://www.forsta.com/ | - Passive sensing: NR - Wearables: NR - EMI: NR | |
| MightyText | | | MightyText  (United States) | | https://mightytext.net/#featurewrap | - Passive sensing: NR - Wearables: NR - EMI: NR | |
| Pingr | | | Pingr Tools LLC  (United States) | | https://pingr.co/ | - Passive sensing: NR - Wearables: NR - EMI: NR | |
| Qualtrics | | | Qualtrics  (United States) | | https://www.qualtrics.com/ | - Passive sensing: NR - Wearables: NR - EMI: NR | |
| Questback | | | Questback  (Norway) | | https://www.questback.com/ | - Passive sensing: NR - Wearables: NR - EMI: NR | |
| REDCap | | | REDCap  (United States) | | https://www.project-redcap.org/ | - Passive sensing: NR - Wearables: NR - EMI: NR | |
| ReTAINE | | | Sanford Health  (United States) | | https://retaine.org/ | - Passive sensing: NR - Wearables: NR - EMI: NR | |
| SoSciSurvey | | | SoSciSurvey  (Germany) | | https://www.soscisurvey.de/en/index | - Passive sensing: NR - Wearables: NR - EMI: NR | |
| SurveyMonkey | | | Momentive  (United States) | | https://www.surveymonkey.com/?ut_source=mp&ut_source2=take-a-tour&ut_source3=header | - Passive sensing: NR - Wearables: NR - EMI: NR | |
| SurveySignal | | | SurveySignal  (United States) | | https://www.surveysignal.com/ | - Passive sensing: NR - Wearables: NR - EMI: yes | |
| Unipark | | | Unipark  (United States) | | https://www.unipark.com/en/survey-software/ | - Passive sensing: NR - Wearables: NR - EMI: NR | |
| WebDataXpress | | | Office of Academic Computing, University of Pittsburgh  (United States) | | https://oac.pitt.edu/services-we-offer/ | - Passive sensing: NR - Wearables: NR - EMI: NR | |
| **User design platforms** | | | | | | | |
| Basic for Android | Anywhere software  (Israel) | | | | https://www.b4x.com/b4a.html | - Passive sensing: accelerometer, gravity, gyroscope, humidity, light, motion, orientation, pressure, proximity, step counter, and temperature - Wearables: NR - EMI: NR | |
| React Native | Meta Platforms, Inc  (United States) | | | | https://reactnative.dev/ | - Passive sensing: accelerometer, gyroscope, magnetometer - Wearables: NR - EMI: NR - Passive sensing: NR | |
| Yapp | Yapp Inc  (United States) | | | | https://www.yapp.us/ | - Wearables: NR - EMI: NR | |

^a^All data were collected from developer websites (provided under the Website column) as of June 2023, and platform capabilities may have changed since the time of data collection.

^b^Developer may reflect a company, institution, or research group.

^c^EMI: ecological momentary intervention. Just-in-time adaptive intervention capabilities were included in the EMI designation.

^d^NR: passive sensing capabilities, wearable integration, or EMI not reported on the developer website.

^e^Capabilities vary for Android and Apple phones.

^f^NA: passive sensing features, wearable integration, or EMI capabilities not available as listed on the developer website.

**Table S2**. EMA platforms developed by researchers for individual use.

| Platform | Developer^a^ | Reference^b^ |
| --- | --- | --- |
| Emotion Sense | University of Cambridge | [37] |
| Healthy UNICT | University of Catania | [38] |
| Imagine Your Mood | University of Groningen, University of Utrecht | [39] |
| mindful moods | Harvard Medical School | [40] |
| Mobiletype | Murdoch Children’s Research Institute | [41] |
| PETE app | Oklahoma State University, Dr Christopher Crick | [42] |
| PSIXPORT | University Autonoma, University Complutense of Madrid | [43] |
| RATE-IT | National Institute of Mental Health, Dr Melissa Brotman | [44] |
| SARA | Harvard University | [45] |
| u-can-act | University of Groningen | [46] |
| Up! | University of Graz | [47] |
| UrbanMind | King’s College London | [48] |

^a^Developer may reflect an institution or research group.

^b^Reference reflects the first publication (to our knowledge) referring to the platform.

Table S1 provides a list of application (app)-, text- and web-based and user design ecological momentary assessment (EMA) platforms, and Table S2 provides a list of EMA platforms developed by research groups for individual use. Our teams created these lists based on our pre-existing experiences with EMA platforms, correspondence with collaborators and colleagues (e.g., word of mouth, social media), and multiple web-based searches. In addition, we created these lists using data extracted from articles included in a systematic review on adherence in pediatric EMA studies (LM Henry et al, unpublished data, 2023). Data were extracted by an independent rater who is a co-author in the present article (EH) and unaffiliated with LM Henry et al. (2023).

Five databases were searched by Alicia A. Livinski, MPH, MA (biomedical librarian at the National Institutes of Health Library with expertise in systematic review searches): PsycNet: PsycINFO & PsycARTICLES (American Psychological Association), PubMed (US National Library of Medicine), Scopus (Elsevier), and Web of Science: Core Collection (Clarivate Analytics). The search strategies incorporated keywords and controlled vocabulary terms (i.e., EMTREE [Embase], MeSH [PubMed], Thesaurus of Psychological Index Terms [PsycNet]) for each concept of interest (i.e., EMA, adolescents or children, psychological symptoms) relevant to the systematic review on adherence in pediatric EMA studies (LM Henry et al, unpublished data, 2023). The search strategy was peer reviewed by a second librarian, Nancy Terry, MLS. Below are details of the search strategy for PubMed.

**Title:** Adherence with Ecological Momentary Assessment Studies in Children and Adolescents with Psychopathology: A Protocol for a Systematic Review and Meta-analysis

**Database:** PubMed/MEDLINE
**Platform:** US National Library of Medicine
**Date Searched:** January 11, 2023

**Date Limits:** None
**Other Limits/Filters:** Language: English; Publication type: Primary research only (excluding reviews, letters, editorials, conference abstracts/proceedings, dissertations, book, book chapters)

| **Set** | **Concept** | **Search Strategy** |
| --- | --- | --- |
| #1 | Ecological Momentary Assessment | ((Ecological Momentary Assessment[Major MeSH] OR "ecological momentary assessment*"[Title/Abstract] OR “ecological momentary intervention*”[Title/Abstract] OR momentary[Title/Abstract] OR “daily diary”[Title/Abstract] OR “daily diaries”[Title/Abstract] OR “electronic diar*”[Title/Abstract] OR “e diar*”[Title/Abstract] OR “e-diar*”[Title/Abstract] OR ediar*[Title/Abstract] OR “digital diar*”[Title/Abstract] OR "Diaries as Topic"[Mesh] OR "event sampling*"[Title/Abstract] OR "experience sampling*"[Title/Abstract] OR “experience-sampling*”[Title/Abstract] OR "real time assessment*"[Title/Abstract] OR “real-time assessment*”[Title/Abstract] OR “just-in-time assessment*”[Title/Abstract] OR “just in time assessment*”[Title/Abstract] OR "short message service*"[Title/Abstract] OR "ambulatory assessment*"[Title/Abstract] OR "text messag*"[Title/Abstract] OR texting[Title/Abstract] OR "Text Messaging"[Mesh] OR “daily prompt*”[Title/Abstract] OR “electronic prompt*”[Title/Abstract] OR “digital prompt*”[Title/Abstract]) NOT sexting[Title/Abstract]) |
| #2 | Digital/Mobile Devices | (cellphone*[Title/Abstract] OR “cell phone*”[Title/Abstract] OR “cellular phone*”[Title/Abstract] OR “cellular telephone*”[Title/Abstract] OR “mobile telephone*”[Title/Abstract] OR “mobile phone*”[Title/Abstract] OR smartphone*[Title/Abstract] OR “palm pilot*”[Title/Abstract] OR “mobile device*”[Title/Abstract] OR “mobile technolog*”[Title/Abstract] OR app[Title/Abstract] OR apps[Title/Abstract] OR “mobile application*”[Title/Abstract] OR computer[Title/Abstract] OR computers[Title/Abstract] OR tablet*[Title/Abstract] OR iPad*[Title/Abstract] OR laptop*[Title/Abstract] OR iphone*[Title/Abstract] OR iPhone*[Title/Abstract] OR Android[Title/Abstract] OR "Smartphone"[Mesh] OR "Cell Phone"[Mesh] OR "Mobile Applications"[Mesh] OR "Computers, Handheld"[Mesh]) |
| #3 | Psychological Symptoms/Psychopathologies | (somatoform[Title/Abstract] OR “Somatoform Disorders”[Major MeSH] OR "eating disorder*"[Title/Abstract] OR “Feeding and Eating Disorders”[Major MeSH] OR "eating patho*"[Title/Abstract] OR "obsessive compulsive disorder*"[Title/Abstract] OR “Obsessive-Compulsive Disorder”[Majr:Noexp] OR "personality disorder*"[Title/Abstract] OR “Personality Disorders”[Major MeSH] OR "self harm*"[Title/Abstract] OR "self-harm*"[Title/Abstract] OR “Self-Injurious Behavior”[Major MeSH] OR “self-injur*”[Title/Abstract] OR “self injur*”[Title/Abstract] OR automutilat*[Title/Abstract] OR “self mutilate*”[Title/Abstract] OR “self-multilat*”[Title/Abstract] OR “self inflicted injur*”[Title/Abstract] OR "sexual problem*"[Title/Abstract] OR "Sexual Dysfunctions, Psychological"[Majr:Noexp] OR “gender dysphori*”[Title/Abstract] OR “gender identity disorder*”[Title/Abstract] OR “psychosexual disorder*”[Title/Abstract] OR “Sexual and Gender Disorders”[Major MeSH] OR anorex*[Title/Abstract] OR Anorexia[Major MeSH] OR "Anorexia Nervosa"[Major MeSH] OR anxiety[Title/Abstract] OR anxieties[Title/Abstract] OR anxious*[Title/Abstract] OR Anxiety[Major MeSH] OR “Anxiety Disorders”[Major MeSH] OR “binge eat*”[Title/Abstract] OR “binge-eat*”[Title/Abstract] OR “Binge-Eating Disorder”[Major MeSH] OR bipolar[Title/Abstract] OR “Bipolar Disorder”[Major MeSH] OR bulim*[Title/Abstract] OR Bulimia[Major MeSH] OR depressi*[Title/Abstract] OR Depression[Major MeSH] OR “Depressive Disorder”[Major MeSH] OR dysthymi*[Title/Abstract] OR “Dysthymic Disorder”[Major MeSH] OR fear*[Title/Abstract] OR Fear[Major MeSH] OR hypomani*[Title/Abstract] OR Mania[Major MeSH] OR internaliz*[Title/Abstract] OR mania*[Title/Abstract] OR manic[Title/Abstract] OR panic[Title/Abstract] OR panics[Title/Abstract] OR panicked[Title/Abstract] OR Panic[Major MeSH] OR “Panic Disorder”[Major MeSH] OR phobi*[Title/Abstract] OR “Phobic Disorders”[Major MeSH] OR suicid*[Title/Abstract] OR Suicide[Major MeSH] OR trauma*[Title/Abstract] OR "thought disorder*"[Title/Abstract] OR paranoi*[Title/Abstract] OR “Paranoid Disorders”[Major MeSH] OR psychosis[Title/Abstract] OR psychotic[Title/Abstract] OR “Psychotic Disorders”[Major MeSH] OR schizo*[Title/Abstract] OR Schizophrenia[Major MeSH] OR "anger control"[Title/Abstract] OR “anger management”[Title/Abstract] OR "attention deficit*"[Title/Abstract] OR “Attention Deficit and Disruptive Behavior Disorders”[Major MeSH] OR "attention problem*"[Title/Abstract] OR "behavior problem*"[Title/Abstract] OR "behaviour problem*"[Title/Abstract] OR “Problem Behavior”[Major MeSH] OR "behavioral development*"[Title/Abstract] OR "behavioural development*"[Title/Abstract] OR "behavioral outcome*"[Title/Abstract] OR "behavioural outcome*"[Title/Abstract] OR "behavioral well-being"[Title/Abstract] OR "behavioural well-being"[Title/Abstract] OR "behavioral wellbeing"[Title/Abstract] OR "behavioural wellbeing"[Title/Abstract] OR "disruptive behavior*"[Title/Abstract] OR "disruptive behaviour*"[Title/Abstract] OR "intermittent explosive disorder*"[Title/Abstract] OR “impulse control disorder*”[Title/Abstract] OR “Disruptive, Impulse Control, and Conduct Disorders”[Major MeSH] OR "oppositional defiant disorder*"[Title/Abstract] OR aggress*[Title/Abstract] OR Aggression[Major MeSH] OR antagonistic[Title/Abstract] OR antisocial[Title/Abstract] OR “anti-social”[Title/Abstract] OR “anti social”[Title/Abstract] OR “Antisocial Personality Disorder”[Major MeSH] OR “conduct disorder*”[Title/Abstract] OR “Conduct Disorder”[Major MeSH] OR disinhibit*[Title/Abstract] OR externaliz*[Title/Abstract] OR hyperactiv*[Title/Abstract] OR hyperkine*[Title/Abstract] OR impulsiv*[Title/Abstract] OR “Impulsive Behavior”[Major MeSH] OR inattenti*[Title/Abstract] OR irritab*[Title/Abstract] OR sociopath*[Title/Abstract] OR “substance use*”[Title/Abstract] OR “substance abuse*”[Title/Abstract] OR addict*[Title/Abstract] OR “drug dependenc*”[Title/Abstract] OR “Substance-Related Disorders”[Major MeSH] OR "communication disorder*"[Title/Abstract] OR “Communication Disorders”[Major MeSH] OR "coordination disorder*"[Title/Abstract] OR "developmental delay*"[Title/Abstract] OR "developmental disorder*"[Title/Abstract] OR “development disorder*”[Title/Abstract] OR “developmental disabilit*”[Title/Abstract] OR “Developmental Disabilities”[Major MeSH] OR "fluency disorder*"[Title/Abstract] OR "intellectual disab*"[Title/Abstract] OR “intellectual impairment*”[Title/Abstract] OR “Intellectual Disability”[Major MeSH] OR "language disorder*"[Title/Abstract] OR “Language Disorders”[Major MeSH] OR “Language Development Disorders”[Major MeSH] OR “language development disorder*”[Title/Abstract] OR “language developmental disorder*”[Title/Abstract] OR “developmental language disorder*”[Title/Abstract] OR “language disabilit*”[Title/Abstract] OR "learning disorder*"[Title/Abstract] OR “learning disabilit*”[Title/Abstract] OR “Learning Disabilities”[Major MeSH] OR "motor disorder*"[Title/Abstract] OR “motor dysfunction*”[Title/Abstract] OR “Motor Disorders”[Major MeSH] OR "movement disorder*"[Title/Abstract] OR “Movement Disorders”[Major MeSH] OR "speech sound disorder*"[Title/Abstract] OR “Speech Sound Disorder”[Major MeSH] OR “Speech Disorders”[Major MeSH] OR “speech disorder*”[Title/Abstract] OR “articulation disorder*”[Title/Abstract] OR autis*[Title/Abstract] OR “Autism Spectrum Disorder”[Major MeSH] OR “Autistic Disorder”[Major MeSH] OR “Asperger Syndrome”[Major MeSH] OR asperger*[Title/Abstract] OR “neurodevelopmental disorder*”[Title/Abstract] OR “neuro-developmental disorder*”[Title/Abstract] OR “Neurodevelopmental Disorders”[Major MeSH] OR stutter*[Title/Abstract] OR Stuttering[Major MeSH] OR tic[Title/Abstract] OR tics[Title/Abstract] OR Tics[Major MeSH] OR Tourette*[Title/Abstract] OR “Tourette Syndrome”[Major MeSH] OR "posttraumatic stress disorder*"[Title/Abstract] OR “post-traumatic stress disorder*”[Title/Abstract] OR “post traumatic stress disorder*”[Title/Abstract] OR “Stress Disorders, Post-Traumatic”[Major MeSH] OR "mental disorder*"[Title/Abstract] OR “Mental Disorders”[majr:noexp] OR "mental illness*"[Title/Abstract] OR "psychological distress*"[Title/Abstract] OR “Psychological Distress”[Major MeSH] OR "psychological impair*"[Title/Abstract] OR “distress syndrome*”[Title/Abstract] OR "psychological symptom*"[Title/Abstract] OR psychopatho*[Title/Abstract] OR Psychopathology[Major MeSH]) |
| #4 | Population Group | (adolescen*[Title/Abstract] OR child[Title/Abstract] OR children[Title/Abstract] OR teen*[Title/Abstract] OR boys[Title/Abstract] OR boy[Title/Abstract] OR girls[Title/Abstract] OR girl[Title/Abstract] OR youth[Title/Abstract] OR youths[Title/Abstract] OR "Adolescent"[Mesh] OR "Child"[Mesh:Noexp]) |
| #5 |  | #1 AND #2 AND #3 AND #4 |
| #6 | Limits Used: Language | #5 AND English[lang] |
| #7 | Limits Used: Publication Type | #6 NOT (letter[Publication Type] OR editorial[Publication Type] OR comment[Publication Type] OR news[Publication Type] OR editorial[Title/Abstract] OR commentary[Title/Abstract] OR "Published Erratum"[Publication Type] OR errata[Title/Abstract] OR erratum[Title/Abstract] OR corrigenda[Title/Abstract] OR corrigendum[Title/Abstract] OR protocol[Title/Abstract] OR protocols[Title/Abstract] OR “meta-analysis”[Title/Abstract] OR “meta-analyses”[Title/Abstract] OR metanalyses[Title/Abstract] OR metanalysis[Title/Abstract] OR metaanalyses[Title/Abstract] OR metaanalysis[Title/Abstract] OR “meta analyses”[Title/Abstract] OR “meta analysis”[Title/Abstract] OR "Review"[Publication Type] OR “systematic review*”[Title/Abstract] OR "Systematic Review"[Publication Type] OR "Meta-Analysis" [Publication Type] OR "Network Meta-Analysis"[Mesh] OR "Review"[Publication Type] OR “integrative review”[Title/Abstract] OR "Case Reports" [Publication Type] OR "case report*"[Title/Abstract] OR "case series"[Title/Abstract] OR "case presentation*"[Title/Abstract] OR "Single-Case Studies as Topic"[Mesh] OR "case stud*"[Title/Abstract] OR "case histor*"[Title/Abstract] OR "single case design*"[Title/Abstract] OR "single-case*"[Title/Abstract] OR "single-case analysis"[Title/Abstract] OR "single cases"[Title/Abstract]) |

**Notes:** The limit for language (English) was applied to the main search using the filters available in PubMed. The keywords were searched in the title and abstract fields in PubMed (i.e., [Title/Abstract]), text field (i.e., [Text Word]), and the controlled vocabulary terms are indicated with [Mesh] or [Major MeSH] where the MeSH terms is the main focus of the article. Terms searched in the [Text Word] field are searched in the title, abstract, keywords, and MeSH fields. Phrases were enclosed in quotation marks to force the searching of the exact terms in order presented. To these results, the search strategy to exclude specific publication types specified in the eligibility criteria was used. No other limits were applied to the search.

**Questions you may be asked by your data security team or Institutional Review Board about your candidate ecological momentary assessment (EMA) platform**

***The National Institute of Mental Health***

1. Where will the data be stored?
2. If the data are stored offsite (outside of [your institution]), is it in a cloud service?
3. If in a cloud service, is the IT application or service FedRAMP compliant?
4. How will the data be encrypted?
5. Does the mobile application (app) allow for two-factor authentication?
6. How will access to the data and the app be limited and to whom?
7. How will the app be secured?

***Boston Children’s Hospital***

1. How is the app downloaded to the participant’s device (app store, installed by sponsor, etc.)?
2. Will data be stored on the participant’s device for any period of time?
3. How is the app secured on the device? Is a password or personal identification number (PIN) for the app required?
4. Will the app be able to access other device functionality such as Location, Contacts, Notifications, Microphone, Camera, etc.?
5. When data is transmitted from the device, where are all of the locations it will reside (even temporarily)?
6. Does the app allow for a wipe of information?
7. How will protected health information (PHI) be stored?
8. Is there an executed Service Agreement? Is there an end-user license agreement (EULA)?
9. Where will PHI and non-PHI data be stored at all points of the study (during collection, during analysis, long term storage, etc.)?
10. Does anyone outside of [your institution] have access to the data?
11. What will happen to the electronic data when the study is completed?
